# Revisiting redox-driven pathways of tin cycle from source to economic deposit

**DOI:** 10.1038/s41598-025-21389-5

**Published:** 2025-10-03

**Authors:** Julie Anne-Sophie Michaud, Christian Schmidt, Maria Aleksandrovna Naumova

**Affiliations:** 1 Institute of Earth System Sciences, Leibniz University, Hannover, Germany; 2 GFZ German Research Centre for Geosciences, Potsdam, Germany; 3 Deutsches Elektronen-Synchrotron DESY, Notkestr. 85, 22607 Hamburg, Germany

**Keywords:** Tin ore deposit, Redox indicator, Tin oxidation state, Magmatic-hydrothermal transition, XANES, Sn k-edge, Geochemistry, Geology, Mineralogy, Petrology

## Abstract

Tin ore deposits are the result of a complex interplay of crustal processes and intensive parameters to enrich tin by a factor of more than 1000. The model for magmatic-hydrothermal tin ore formation, established for more than three decades, dictates that reducing conditions and redox reactions play a crucial role for tin transport and concentration. Yet, no study has systematically documented the tin oxidation state in crustal rocks involved in tin-ore formation, which was mainly due to analytical challenges. This study shows that X-ray Absorption Near Edge Structure (XANES) spectroscopy allow direct assessment of the tin oxidation state at concentrations as low as 30 ppm. Here, we report the oxidation state of tin for a wide range of reference materials and natural rock samples, often for the first time. Our findings that Sn^IV^ is dominant in crustal metamorphic, magmatic and hydrothermal rocks suggest that redox conditions play a smaller role in tin-ore formation than previously postulated, with redox reactions being significant, but not essential, during granitic magma generation and crystallization. Moreover, this study demonstrates that tin can be used as new redox indicator to help solving major questions such as redox conditions in the continental crust or Earth’s redox evolution.

**Supplementary Information:**

The online version contains supplementary material available at 10.1038/s41598-025-21389-5.

## Introduction

**Fig. 1:**
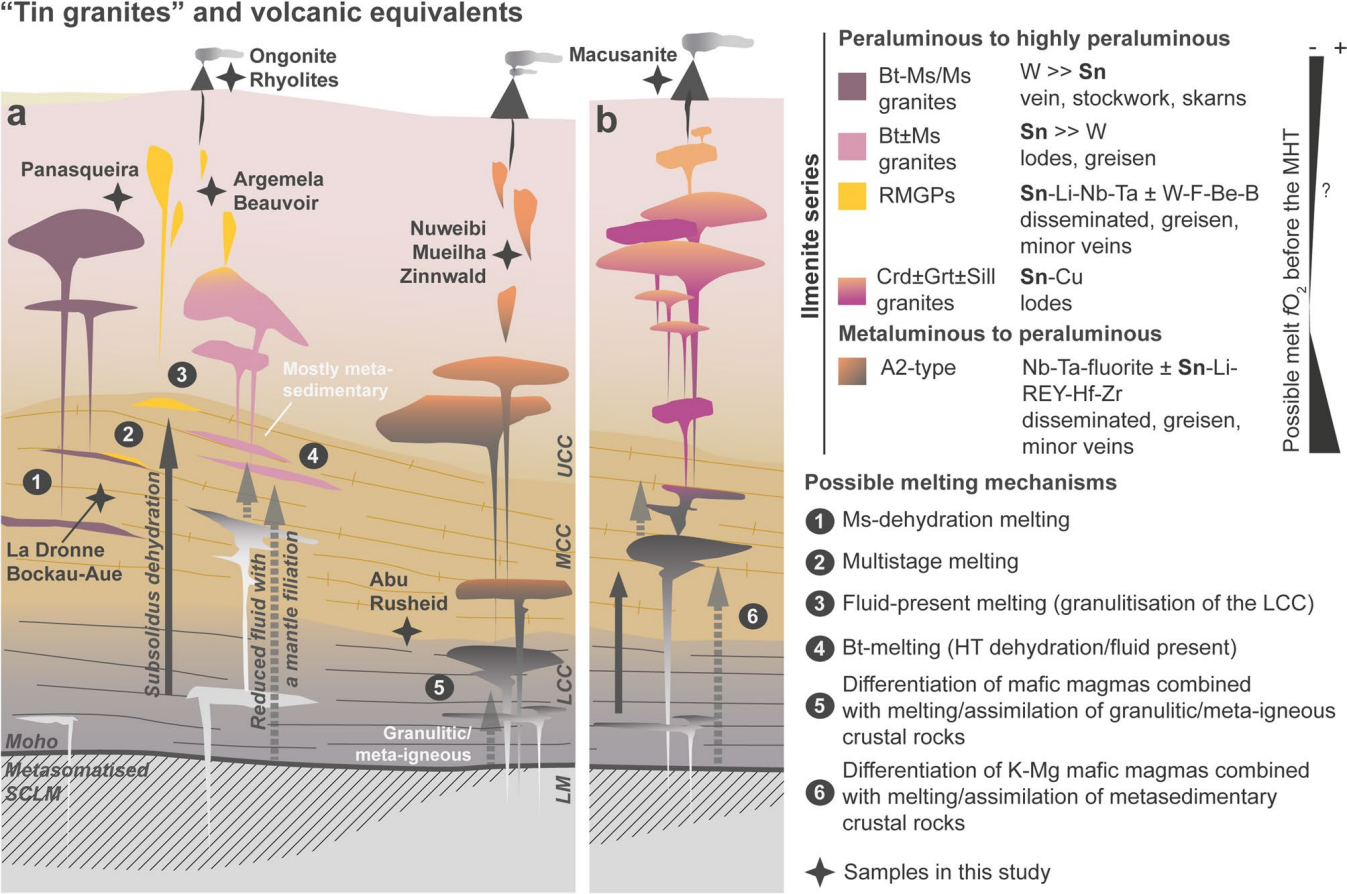
Sketch illustrating the variety of “tin granites” and volcanic equivalents in relation with the main commodities, mineralisation styles and possible melting mechanisms. Typical “tin granites” from late to post-collisional environment of (a) ancient continent-continent orogenies and (b) Andean-type orogenies. Note that melting mechanisms − (1)^7^, (2)^54^, (3)^55^, (4)^55^, (5)^9^ and (6)^47^ - strongly influenced by the geodynamical setting, might significantly control the initial *f*O_2_ of granitic melts/magmas (≤ NNO; e.g., internally vs. externally buffered). UCC, MCC, LCC: upper, middle, lower continental crust; LM: lithospheric mantle; SCLM: sub-continental lithospheric mantle; MHT: magmatic-hydrothermal transition; HT: high temperature. See text for discussion.

**Fig. 2:**
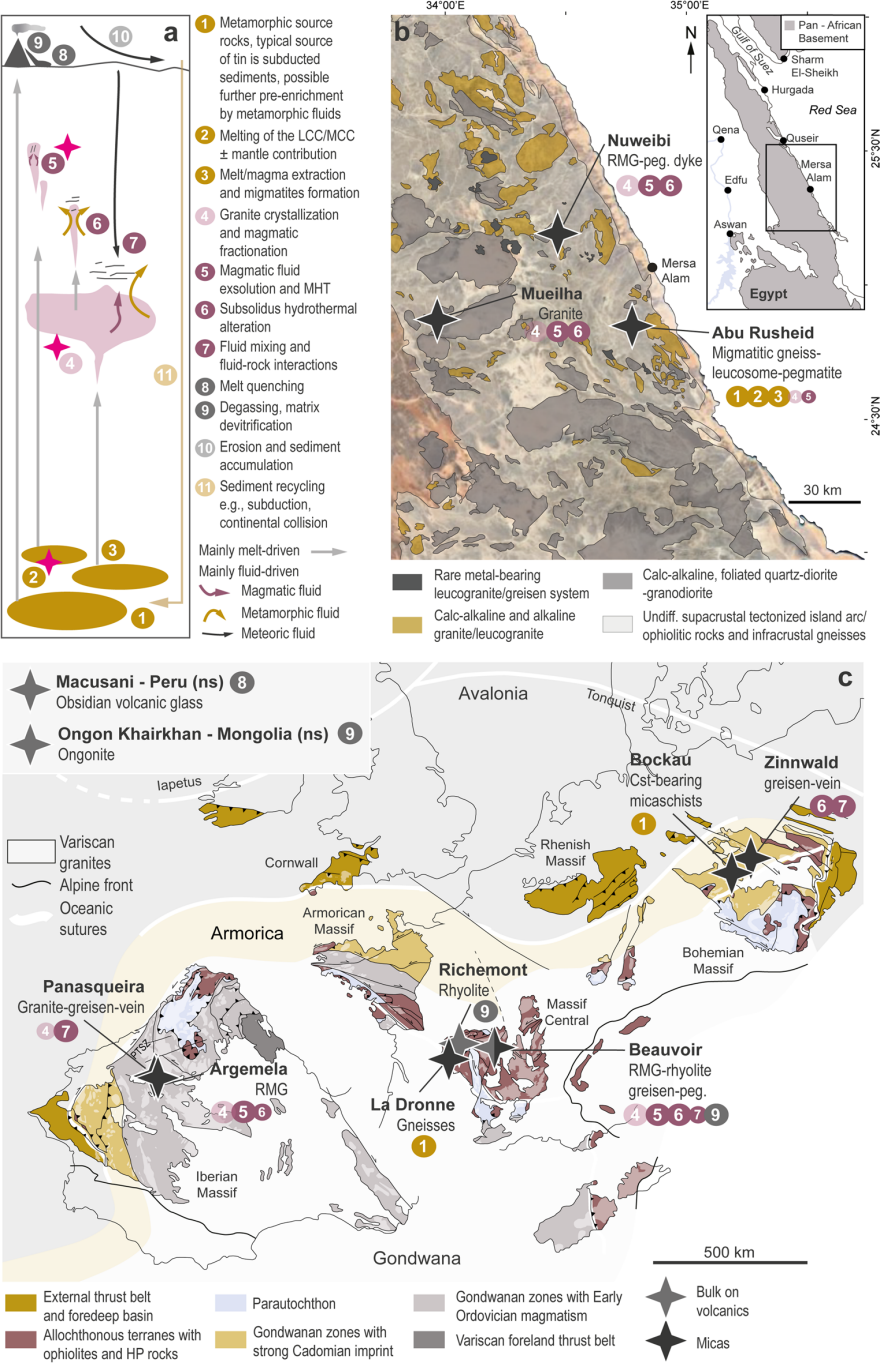
Processes in the geochemical cycle of tin (**a**) and sample locations (**b,c**). (**a**) Pink stars: major redox control in tin cycle. (**b**) Simplified geological map of the Central Eastern Desert of Egypt modified after^57^ (and references therein) showing sample locations and associated processes. (**c**) Sketch of the Variscan belt of Western Europe representing the main units and geodynamic features modified after^58^ (and references therein), with sample locations and associated processes. RMG: rare metal granite; peg.: pegmatite; Cst: cassiterite; ns: not shown on map; LCC/MCC: lower/middle continental crust; MHT: magmatic-hydrothermal transition. The different symbol sizes representing involved processes in each location denote their approximate relative importance. Maps were vectorized and modified using Adobe Illustrator 2024 (version 28.0; Adobe Inc., San Jose, CA, USA; Available at: https://www.adobe.com/products/illustrator.html) and the QGIS Geographic Information System (version 3.34; Available at: https://qgis.org). Permissions to modify the figures have been granted (Licence ID: 1639027-1 and 1639027-2)

Tin is present as a trace element in rock forming minerals at all levels of the continental crust and is a main commodity in magmatic-hydrothermal deposits, mainly found associated with ilmenite-series granites (Fig. 1). To unravel processes governing tin transport and concentration into economic deposits - and to guide exploration and discovery of new resources - numerous studies have focused on tin. The intricate interplay of crustal processes (Figs. 1 and 2a) and intensive parameters - such as oxygen fugacity (*f*O₂), pressure, and temperature - is widely acknowledged, however, critical questions persist. In particular, redox conditions are assumed^1,2^ to critically control tin’s behaviour but comprehensive studies systematically determining the oxidation state of tin in crustal rocks relevant to the tin cycle remain notably absent.

The established model for tin ore deposit formation dictates that ilmenite-series tin-specific granites derive from highly fractionated partial melts/magmas of reduced metasediments with near-crustal average tin content, and that tin is transported in reduced granitic peraluminous magmas and exsolved hydrothermal fluids as Sn^II^ followed by oxidation and precipitation mostly as cassiterite, SnO_2_^2^. However, there is growing evidence that there is more than one path to a hydrothermal tin deposit. Firstly, tin-enriched metasediments exist that are likely protoliths of tin-specific granites in tin provinces^4–8^ and mantle filiation has been proposed as an additional factor controlling redox conditions in the source area^9,10^ (external redox buffers; e.g., Fig. 1). Secondly, in granitic melts, the Sn^II^-Sn^IV^ transition is at log *f*O_2_ of + 1 to + 2 relative to the quartz-fayalite-magnetite buffer (QFM) and the solubility of Sn^II^ is much higher than that of Sn^IV^^11^, which indicates that magmatic cassiterite should not crystallize in ilmenite-series granites under reducing conditions. Yet, magmatic Sn^IV^-minerals (particularly cassiterite, less common wodginite and ixiolite) are frequently observed in rare metal granites and pegmatites (RMGPs)^13^, and even in less fractionated ilmenite-series tin-granites (e, g., ~ 80 ppm Sn^14^) and ongonite (e.g., ∼ 40 ppm Sn^15^). Thirdly, a central role of low *f*O_2_ seems obvious given that Sn^II^ is incompatible and thus strongly prefers the granitic melt over restite minerals^16^. However, magmatic cassiterite in granites and pegmatites indicates rather oxidised conditions, which is further supported by the presence of primary CO_2_-rich inclusions without graphite^17^ and by the much lower solubility of Sn^IV^ in granitic melts compared to that of Sn^II^ ^11^. It has been argued that oxidation can occur earlier in the course of granite crystallization and the magmatic-hydrothermal transition^18^ (MHT). If no oxidation took place in the granitic melt, the highly incompatible Sn^II^ should remain in the melt or be incorporated into micas - possibly substituting for K or through other mechanisms - which has not been demonstrated yet. Lastly, Sn^IV^-chloride species seem more abundant than Sn^II^-chloride complexes in hydrothermal fluids at *f*O_2_ ≥ NNO (nickel-nickel oxide buffer)^17,19,20^. This is in very good agreement with compositions of primary fluid inclusions in cassiterites from hydrothermal vein/greisen deposits, which indicate precipitation at *f*O_2_ between near NNO to magnetite-hematite^17^ and thus at much more oxidised conditions than inferred for tin greisens^2^.

In its cycle from a source to a deposit (Fig. 2a), tin is almost exclusively tetravalent at the first stages (continental erosion and transport to continental margin) and predominantly tetravalent at the end (magmatic or hydrothermal tin mineralisation^6)^. Predominance of divalent tin is plausible at high temperatures at which reduced assemblages are more stable at a given *f*O_2_. If so, at least two redox reactions should take place if redox partners are available, from Sn^IV^ to Sn^II^ at some time during accretion, subduction, and partial melting, and from Sn^II^ to Sn^IV^ to form hydrothermal mineralisation. For direct assessment of the role of redox reactions during the geochemical cycle of tin, it is crucial to determine the oxidation state of tin in different geological environments. This is difficult because there are very few techniques that can be used to distinguish Sn^II^ and Sn^IV^ particularly if present as a trace element. Also, due to its incompatibility, Sn^II^ can substitute only in few sites in rock-forming silicates, likely for K in phyllosilicates, which are the most widespread carriers of tin in the continental crust, with concentrations ranging from a few ppm to hundreds of ppm^13,21^. To our knowledge, the actual oxidation state of tin at such concentrations has never been determined in natural samples such as metasediments or granites. In principle, the shift in the energy of the Sn K-edge at about 29,200 eV in XANES spectra provides direct qualitative information on tin oxidation state^22^. Here, we show, for the first time, that this technique can be successfully used to explore the tin oxidation state in micas and rocks from different geological environments relevant to the tin cycle (e.g., Fig. 2a). The redox-driven pathway of tin to form economic deposit is revisited and the applicability of tin as a new redox indicator is discussed.

### “Tin-granites”: short overview and redox considerations

Fundamentally, the term “tin-granite” was first introduced by Humboldt^23^ to refer to the close association between tin deposits and specific granites. After more than 200 years, this term is still sporadically used in the literature but as emphasised in recent studies^2,24^, the definition behind is rather arbitrary and tin levels of enrichment and mineralisation style vary significantly among granite types in different geodynamical environments (see Fig. 1 for a modest attempt at synthesis). In late to post-collisional environments of ancient continent-continent orogenies (e.g., the Western Europe Variscan belt^13,25–27^, the Nanling Region^28^, Eastern Desert of Egypt^29^, tin deposits are generally associated with two micas/muscovite granites (W > > Sn), biotite ± muscovite granites (Sn > > W), RMGPs (Sn-Li-Nb-Ta ± W) and ± evolved A2-type granites (Nb-Ta ± Sn; Fig. 1a). In extensional environments of Andean-type orogenies such as the Central Andean Tin Belt (e.g., San Rafael^30^, tin is mainly associated to Crd ± Grt granites (Sn-Cu; Fig. 1b). Most tin granites, including the ones considered in this study (Fig. 1, Table S-1), belong to the “so-called” ilmenite series (*f*O_2_ ≤ NNO^31^), redox conditions being based on the presence and stability of magmatic ilmenite. Yet, ilmenite is not always present (e.g., RMGPs^13)^, its mere presence does not imply reduced conditions, and geodynamic processes may exert a primordial control on granitic melt generation and acquisition of their primary geochemical signatures^2,9,24^. During the typical geodynamic evolution from collisional to late/post-orogenic settings, redox conditions are likely to shift - from being internally buffered (e.g., by C-bearing crustal rocks) to externally buffered (e.g., by reduced fluids or mantle-derived melts), as mantle involvement increases due to litospheric delamination and mantle upwelling. Ultimately, tin preconcentration in granitic systems results from the interplay between redox conditions and the aluminum saturation index of the melt, which governs the solubility of SnO₂^11^ (Fig. 1). For example, Macusani obsidian glasses and possibly granites related to the San Rafael tin world-class deposit may result from extremely reducing conditions (i.e., influence of reduced mafic magmas) as indicated by low Fe^III^/Fe^II^-ilmenite and magmatic biotite^10,18,30^. In comparison, rare data on ilmenite from A2-type granites such as Abu Dabbab^32^ (i.e., relatively high Fe^III^/Fe^II^ and high MnO) might indicate higher initial *f*O_2_ of the melt and their weakly peraluminous character, mainly inherited from source rocks, limit tin preconcentration. In addition, it is to be expected that granitic melt initial redox will affect the behaviour of tin during the magmatic-hydrothermal transition. Despite some existing research and development on the topic^18,33–35^, it remains difficult to precisely quantify the relative *f*O_2_ of granitic melts because: (i) ilmenite is not always present and when it is, its composition and redox partners are rarely available; (ii) one^18^ of the very few studies investigating the relationship between *f*O_2_ and Fe/(Fe + Mg) in mica - crystallizing in equilibrium with weakly to highly peraluminous melts - found no systematic correlation between the two parameters. This suggests that Fe might fail as a redox indicator in micas from evolved, Fe-Mg-poor granitic systems; (iii) the accuracy of a recently developed method consisting in using trace elements in zircon as an oxybarometer^35^ diminishes drastically for peraluminous to highly peraluminous composition, which are underrepresented in the calibration dataset. In addition, zircon is often absent in RMGPs, or it may be strongly metamict and/or heavily modified due to subsolidus alteration in granitic systems (e.g., Fig. S-1). As a result, zircon does not reliably record redox changes during crystallization and the MHT. Thus, it is of general interest to define new redox indicators for evolved granitic systems.

## Samples relevant to the tin cycle

Because major crustal processes responsible for tin ore deposit formation involve metamorphic, magmatic rocks and hydrothermal fluids, it is crucial to investigate a broad range of relevant geological samples (Figs. 1 and 2). Most samples stem from two tin provinces, the Central Eastern Desert of Egypt (Fig. 2b) and the Western European Variscan belt (Fig. 2c; see Tables S-1 and S-2 for more detailed sample descriptions and references). To assess tin oxidation state in potential source rocks for peraluminous melt generation, we studied micaschists from the Bockau-Aue region, Erzgebirge, Germany (Fig. 2c), an orthogneiss from the French Massif Central (FMC) (Fig. 2c) and two migmatitic gneisses from Abu Rusheid, Egypt (Fig. 2b). The signature of granitic melts generated by partial melting and fractionation was studied based on a leucosome from Abu Rusheid (Egypt), rhyolite samples from Richemont and Beauvoir (FMC), ongonite from the type locality Ongon-Khairkhan (Mongolia) and obsidian glass from Macusani (Peru), a volcanic equivalent of peraluminous rare-metal-bearing granites. Several magmatic-hydrothermal systems in Western Europe and Egypt were selected to reflect the variable contributions of magmatic differentiation, MHT and subsolidus hydrothermal alteration (Figs. 2b, c). Mica samples from the highly peraluminous rare-metal granites of Argemela, Central Portugal (border aplitic/pegmatitic unit and granitic facies) and Beauvoir, FMC (granitic facies B1 and B2, greisen and pegmatitic pocket), were analysed to evaluate if changes in tin oxidation state occurred in the course of early magmatic-hydrothermal processes. Samples from Nuweibi, Egypt (granitic facies and pegmatitic vein), Abu Rusheid, Egypt (pegmatite) and Mueilha, Egypt (border unit and red granite) are representative for weakly peraluminous systems with a moderate to high hydrothermal imprint. Samples from Panasqueira, Central Portugal (granite, greisen and vein selvage) and Zinnwald, Erzgebirge, Germany (greisen and vein), were analysed to look at the effect of strong hydrothermal overprint including fluid mixing, fluid/rock interaction and subsolidus alteration. As emphasised in the previous section, all granitic systems in this study belong to the ilmenite series (*f*O_2_ ≤ NNO^31^) and are representative of different geodynamical context, melting mechanisms and possibly, variable melt initial *f*O_2_ ≤ NNO (e.g., Figs. 1 and 2, Table S-1).

To determine tin oxidation state in natural samples, it seems fundamental to evaluate their degree of preservation and their significance (see Table S-1 for possible significance of tin redox in each sample). Most samples measured in this study were studied before with data and interpretation published (see Table S-1). For example, the magmatic, magmatic-hydrothermal and subsolidus evolution of Argemela has been in part defined based on the study of mica texture (Fig. S-2a-b), stable isotopes (O, H) and major and trace element signatures^13^, demonstrating that the different stages are preserved and no external meteoric or metamorphic fluid is involved in their crystallization. Other studies demonstrated that lepidolite in Beauvoir (B1-B2; e.g., Fig. S-2c) is magmatic while muscovite occurring as rims (e.g., Fig. S-2c) or replacing magmatic lepidolite result from magmatic-hydrothermal and later subsolidus processes with the involvement of meteoric fluids, respectively^36,37^. In addition, the composition of magmatic lepidolite could be reproduced in crystallization experiments^38^ demonstrating their pristine character. In our opinion, the pristine character of Macusani obsidian glass is no longer to be demonstrated^10^ (Fig. S-2d). The sections from Bockau-Aue (Erzgebirge) and Ongon-Khairkhan (Mongolia) were prepared from the interior of larger samples and showed no sign of weathering (e.g., black biotite, no hint of a bronze tint). Magmatic micas in Mueilha samples are variably overprinted and replaced by muscovite under subsolidus conditions (see Fig. S-2e for an extreme example) indicating that their tin redox signature should reflect coupled dissolution-precipitation resulting from fluid-driven processes (see also Table S-1)^39^. The few samples that are not yet part of a publication (Abu Rusheid) were investigated as part of ongoing research and checked for secondary alteration, unrelated to processes relevant to the tin cycle. Primary mica in Abu Rusheid seems relatively well preserved (e.g., Fig. S-2f) although signs of fluid circulation and transformation under subsolidus conditions are present. In preserving tin oxidation state in relation to a certain process, time is another factor to consider as Neo-Proterozoic samples from Egypt have theoretically greater chance to be modified than Macusani glasses. Though, this can only happen provided that a redox partner is available after the high temperature magmatic-hydrothermal activity and should be accompanied by textural/chemical changes, not observed in our samples.

### X-ray absorption spectroscopy

Tin K-edge X-ray absorption fine structure (XAFS) spectra were collected at beamline P64 of the PETRA III storage ring at Deutsches Elektronen-Synchrotron (DESY) in Hamburg. A Si(311) double-crystal monochromator was used for energy selection^40^. Samples were affixed to the sample holder with sticky Kapton tape and spectra were measured simultaneously in transmission and fluorescence modes in the − 150 to 400 eV energy range around the Sn K-edge. X-ray fluorescence was registered with a Passivated Implanted Planar Silicon (PIPS) detector. Fluorescence spectra were utilised if they exhibited superior quality compared to transmission spectra and showed no signs of self-absorption. Simultaneously with the samples, a tin foil was measured to account for possible deviation over time, and the energy position of the maximum of the first derivative of its absorption coefficient was set to 29200.4 eV (in this case, the energy at ½ edge jump intensity is 29197.9 eV). Consequently, all spectra were calibrated to this value. All XANES spectra were processed using the Athena^41^ software package and were normalised for absorbance following standard methods. A linear background was subtracted from the pre-edge (-150 to -70 eV) resulting in the pre-edge being 0, the edge jump was set to be 1 and post-edge (50 to 350 eV) regions and the spectra were then divided by the value of the edge jump at the absorption edge. All presented spectra are normalised but not flattened, resulting in the post-edge regions not oscillating around 1 as often observed for XANES data. This has no effect on the edge position and shift.

### Applicability of the method to natural samples

**Fig. 3:**
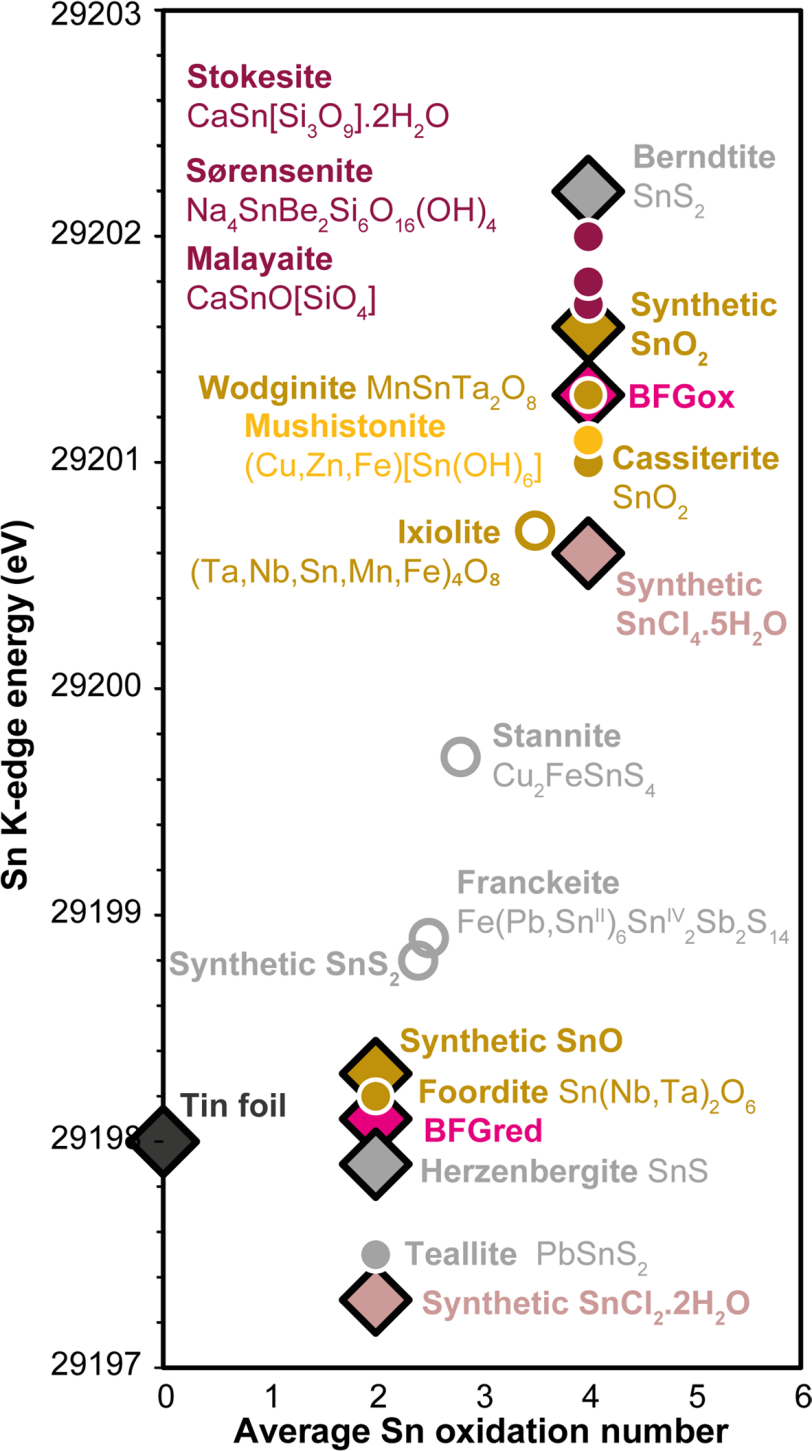
K-edge energies at 50% of the edge jump intensity of the normalised XANES spectra as function of the average tin oxidation number. Filled symbols: known tin oxidation number; diamonds: selected reference substances for metallic tin (dark grey), oxides (brown), silicate glasses (fuchsia), sulphides (grey), and chlorides (light pink); circles: other tin-bearing minerals with known oxidation number; open symbols: unknown average tin oxidation number (calculated based on the K-edge energies of the reference sulphides and oxides). Burgundy symbols: silicates, yellow symbol: hydroxide.

**Fig. 4:**
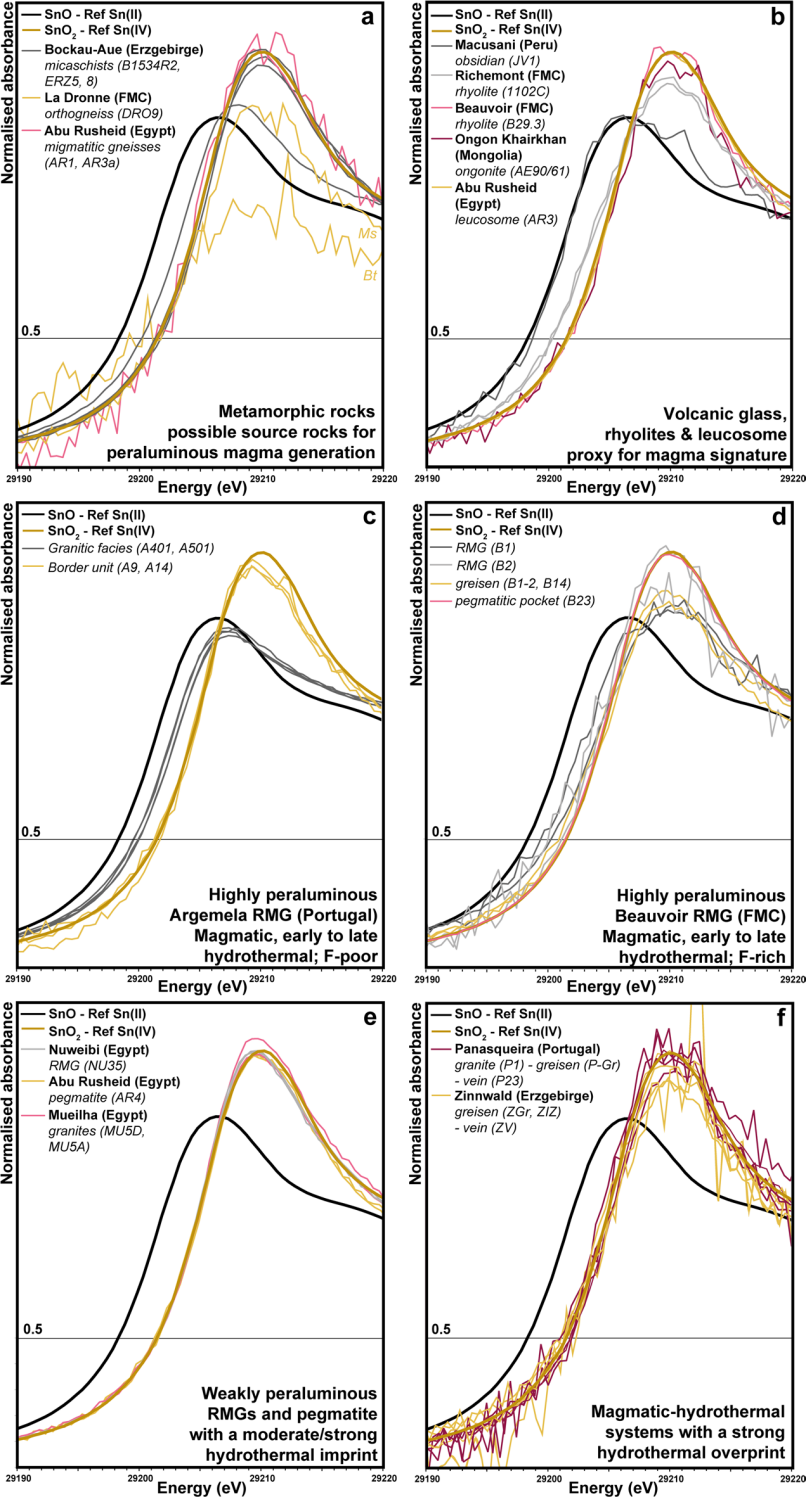
Normalised XANES spectra at the Sn K-edge of all investigated samples from metamorphic rocks (**a**), volcanic glass, rhyolites and a leucosome (**b**), the Argemela rare-metal granite (RMG) (**c**), the Beauvoir RMG (**d**), granites and pegmatites from Egypt (**e**), and magmatic-hydrothermal W-Sn deposits (**f**). FMC: French Massif Central. Ms: muscovite; Bt: biotite. Spectra were obtained on mica except for the Macusani sample (glass) and the rhyolite/ongonite samples (bulk).

The method of using the shift in the energy of the Sn K-edge on XANES spectra to determine tin oxidation state has been tested^22^ on hydrous haplogranitic silicate glasses synthetised at different *f*O_2_. Although this study proved the suitability of the method on reference materials, an unresolved discrepancy remained between the redox conditions of glass synthesis and measured oxidation state of tin in the peralkaline and peraluminous glasses. Here, we analysed a large range of substances with known oxidation states, different tin coordination and next neighbours (O, Cl, S, OH) to study in detail the applicability of the method to natural samples with unknown tin oxidation state (Figs. 3, S-3, S-4; Table S-3). To evaluate redox state, the “E0” energy for each reference and sample normalised spectra was measured using the “half way” method, i.e., the energy at 50% of the absorption edge jump intensity. The analyses of the substances with known tin oxidation state showed that determination of the energy of the Sn K-edge is a robust technique to distinguish Sn^II^ and Sn^IV^, which confirms previous conclusions^22^. The Sn^IV^ K-edge energies are by about 2 to 4 eV higher than those of Sn^II^ (Figs. 3, S-3, S-4, Table S-3) giving good confidence in the determination of the predominant tin oxidation state in natural rocks and micas. However, there is considerable variability in the K-edge energies in the six Sn^II^ and the ten Sn^IV^ references (Fig. 3; Tables S-3). Observed absorption edges of SnO and cassiterite are by 2.9 and 3.2 eV lower than reported^22^ mainly because different X-ray energies were used for calibration. The difference of 0.6 eV between the K-edge energies of synthetic SnO_2_ and cassiterite may be taken as estimate for the uncertainty of the method (Fig. 3). Values for Sn^II^ and Sn^IV^ chloride hydrate are by about 1 eV lower than those of the oxide references SnO and SnO_2_, and the Sn K-edge energy of sulphides appears to increase more with oxidation number than those of oxides, silicate glasses, or chlorides (Fig. 3). All analysed samples with unknown average tin oxidation number (franckeite, synthetic SnS_2_, stannite, ixiolite) show Sn K-edge energies between that of the reference Sn^II^ and Sn^IV^ sulphides and oxides, which indicates that they contain mixed tin oxidation states (Fig. 3). Details on variations of the peak height of the edge-jump in XANES spectra are available in the Supplemental Material (see also Fig. S-5). Overall, tin oxidation states obtained in reference substances enable the method to be validated and applied to 31 natural micas and rocks for concentrations as low as 30 ppm (Fig. 4a-f; Table S-4).

**Fig. 5:**
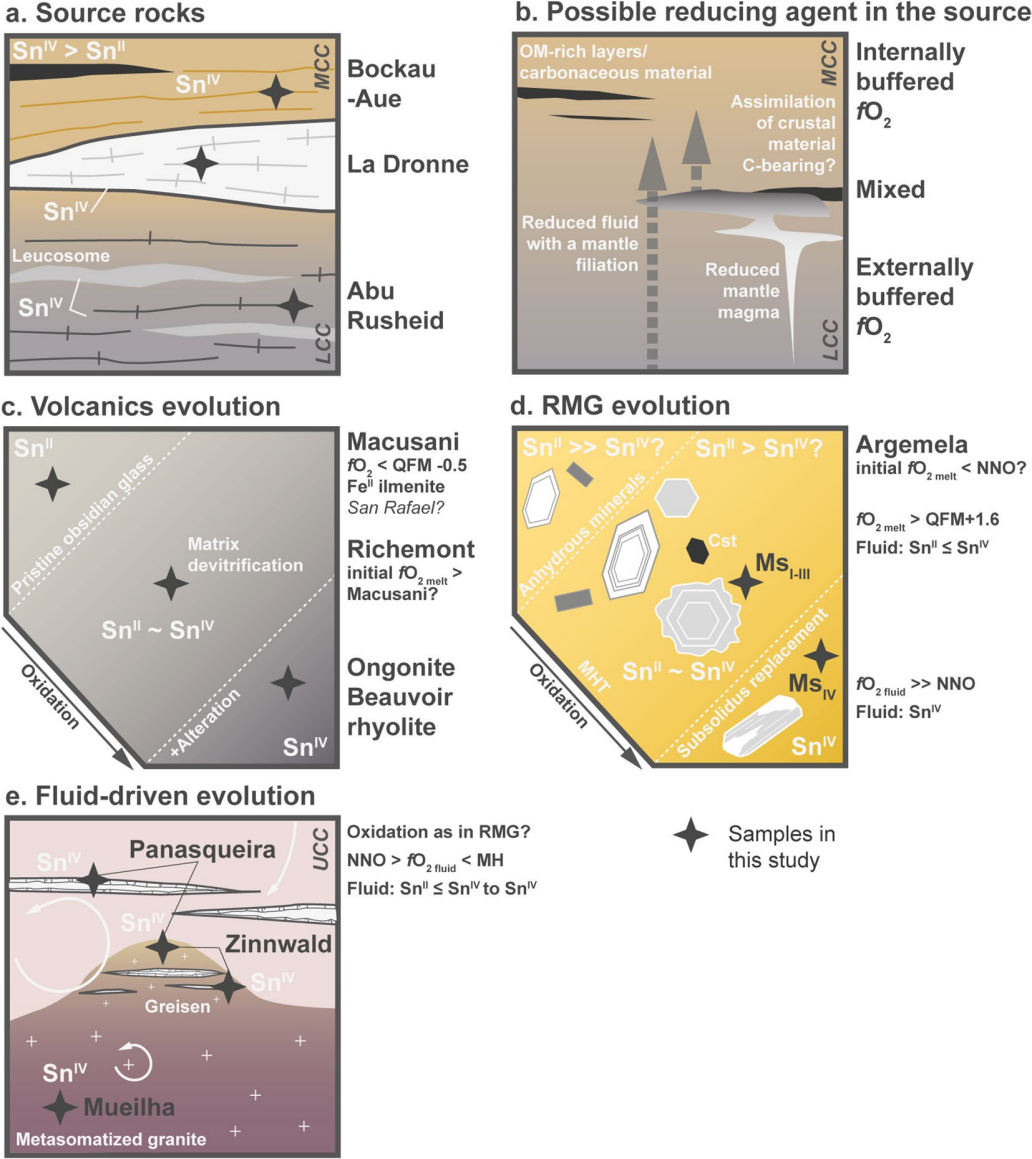
Illustration sketch summarising tin redox state in various geological environments based on the results of this study and main redox evolutions. UCC, MCC and LCC as in Fig. 1; OM: organic matter. Colours similar to Fig. 1. See text for discussion.

## Results

Here, we successfully applied the method for the first time to natural samples from various geological environments (Fig. 4a-f; Table S-4; see Supplemental Material for more details about reference materials and samples; Figs S-3, S-4; Table S-3). Tetravalent tin is predominant over divalent tin in most samples regardless of location or environment (i.e. metamorphic, magmatic, early and late hydrothermal). Macusani obsidian is the only sample showing only divalent tin with no hint of Sn^IV^ (Fig. 4b). Mixed oxidation states were observed in some samples from Bockau-Aue (Erzgebirge) (Fig. 4a), in the Richemont rhyolite (Fig. 4b), and in magmatic-hydrothermal micas from the granitic facies of Argemela (Fig. 4c) and Beauvoir RMGs (Fig. 4d).

### Tin redox-driven pathways from source to deposit

The predominance of Sn^IV^ in natural samples studied here prompts the question of whether a realistic pathway exists for tin cycling and ore formation at normal crustal *f*O_2_ near NNO. Even at high temperatures, Sn^IV^ will not be reduced if a redox partner is absent. Consequently, the reactions governing tin ore formation must be formulated such that tin transport and precipitation can proceed with and without redox processes. In other words, models need to account for multiple pathways towards tin ore deposits.

The Sn K-edge XANES spectra of the metamorphic rocks indicate, with one exception, only tetravalent tin (Figs. 4a and 5a), regardless of metamorphic grade and age (Table S-1). A mixture of Sn^II^ and Sn^IV^ was observed only in a micaschist from the Garnet Phyllite Unit at Bockau-Aue, Erzgebirge (Figs. 4a and 5a). In this sample, cassiterite-free spots were identified by XANES scans across the thick section, i.e. the XANES spectrum shown in Fig. 4a represents tin incorporated in mica. Even the migmatitic gneiss and leucosomes from Abu Rusheid show no sign of reduction to Sn^II^ (Fig. 4a, b). While acknowledging that a larger sample set is necessary for a fully representative assessment of redox in crustal source rocks, these findings suggests that tin granites can possibly form from source rocks lacking a reducing agent such as carbonaceous material (i.e., *via* internal redox buffering). This seems particularly reasonable for the formation of A2-type granites (Figs. 1 and 4e) for which granulitic or meta-igneous source rocks are favoured (see^42^ for a discussion). Additionally, tin is not the primary commodity in these systems. Although SnO_2_ is much less soluble in granitic melts than SnO, oxidised granitic magmas can still dissolve several tens of ppm of tin and, at higher tin concentrations with increasing fractionation, crystallize magmatic cassiterite. Alternatively, external reducing agents - such as reduced melts or fluids with a mantle filiation (i.e., external redox buffering; Fig. 5b)^43^ - can be involved in the melting reaction. While this is very likely in the Andes (e.g., Macusani^9,10^ and neighbouring tin world class deposit San Rafael^30^), it remains to be demonstrated for other geodynamic settings^9^. Variations in redox conditions in the source region - whether internally or externally buffered - driven by geodynamic evolution and melting processes, may exert a key control on tin preconcentration in granitic melts/magmas and ultimately determine if tin is the dominant commodity in resulting ore deposits (Fig. 1).

There are several arguments suggesting that tin granitic melts/magmas are relatively reduced (e.g., ilmenite crystallization, low Fe^III^/Fe^II^, higher SnO_2_ solubility)^10,11,31^. This idea is strengthened by the presence of only divalent tin in Macusani obsidian (Fig. 4b), which supports the conclusion by Pichavant et al.^9,44^. 

The extremely reduced character of Macusani glasses - < QFM-0.5 to account for the presence of Sn^II^ only^11^ - may reflect redox conditions in the source region by external reducing agents (Fig. 5b). Under such reducing conditions, peraluminous granitic melts can carry up to ~ 4wt% “SnO_2_”^11^. This, combined with long-lived magmatic activity in the Central Andean Tin belt, is likely a key factor controlling the unique and never equalled grades in San Rafael^45^. Such exceptional conditions might not be met in other settings and other paths need to be considered. Though, and as emphasised previously, comparing the absolute *f*O_2_ of parental melts/magmas of tin granites is challenging. Other high-grade lodes deposits in late orogenic settings (e.g., Cornwall^26^; Nanling^28^) are commonly associated with ilmenite-bearing biotite granites (Fig. 1) and are thus interpreted to have crystallized at *f*O_2_ ≤ NNO^18^. However, late orogenic rare metal granites (Fig. 1) are more problematic as biotite and ilmenite are generally absent. Relations connecting *f*O_2_, Fe^III^/Fe^II^, and mica composition in equilibrium with weakly to highly peraluminous melts remain experimentally unconstrained (see^33^ for an example in presence of fluid only) and no pristine glass in rare metal volcanics has been found in such context. The mixed state (Sn^IV^> Sn^II^; Fig. 4b) in the Richemont rhyolite can possibly be attributed to partial oxidation during matrix devitrification/degassing (Fig. 5c) while the Sn^IV^ signature of Ongonite and the Beauvoir rhyolite result mainly from intensive metasomatic alteration (Fig. 5c).

If partial granitic melts are relatively reduced ≤ NNO, this raises the question at which stage tin is oxidised to Sn^IV^ to form tin-oxides. Our findings in micas from RMGs at Argemela and Beauvoir (Fig. 4c, d and S-2a, b), which follow a similar evolution, point to redox changes in the course of magma crystallization and the MHT. One would expect that magmatic-hydrothermal micas reflect the reduced signature of the magma, especially in Argemela where fluids are almost exclusively magmatic and fluid-rock interactions are near-absent^13^. In contrast, muscovite, the main mica crystallizing at the magmatic stage and along the MHT in Argemela^13^, is only stable under relatively oxidising conditions^18^ (> QFM + 1.6). Therefore, the mixed oxidation states of tin found in magmatic-hydrothermal micas (Figs. 4c and 5d and S-2a; Table S-4) suggest that (i) Argemela must have undergone oxidation at the magmatic stage and along the MHT, which is in good agreement with early magmatic cassiterite crystallization^13^, (ii) *f*O_2_ was > QFM + 1.6 to account for the fraction of species^11^ and muscovite crystallization^18^, and (iii) magmatic fluids exsolving from the crystallizing magma should be relatively oxidised carrying a significant fraction of Sn^IV^. The latter is further supported by the presence of only tetravalent tin in the border unit at Argemela, where primary magmatic-hydrothermal muscovite underwent subsolidus hydrothermal replacement by Li-bearing micas^13^ (Figs. 4c and 5d and S-2b; Table S-4). A possible mechanism may be “self-oxidation”, e.g., *via*

1 $${{\text{H}}_{\text{2}}}{\text{O }}\left( {{\text{melt}}/{\text{gas}}} \right){\text{ }} + {\text{ 2}}{{\text{F}}^ - }\left( {{\text{melt}}} \right){\text{ }} = {\text{ 2HF }}\left( {{\text{gas}}} \right){\text{ }} + {\text{ }}{{\text{O}}^{{\text{2}} - }}\left( {{\text{melt}}} \right)$$

2 $${\text{S}}{{\text{n}}^{{\text{II}}}}{\text{O }}\left( {{\text{melt}}} \right){\text{ }} + {\text{ }}0.{\text{5}}{{\text{O}}_{\text{2}}} = {\text{ S}}{{\text{n}}^{{\text{IV}}}}{{\text{O}}_{\text{2}}}\left( {{\text{melt}}} \right)$$

(modified after^18^. If so, the activity of F^−^ could determine the extent of oxidation (e.g., F-poor Argemela *vs* high-F Beauvoir) and Sn^IV^/Sn^II^, affecting significantly the mineralising potential of magmatic fluids. If oxidation during crystallization and the MHT cannot be demonstrated in this study for less evolved tin-granites (e.g., cordierite-, muscovite-biotite- or biotite-granites; Fig. 1), such redox pathway has been documented in the literature^34,43^. Yet, using tin oxidation state in mica as a proxy for redox conditions in a granitic melt may raise some questions. One might expect that Sn^II^ and Sn^IV^ would fractionate due to differences in ionic radius, with Sn^IV^ being more readily incorporated into mica structures. However, direct evidence for the specific substitution mechanisms of tin in mica is lacking; current interpretations are largely based on crystallographic considerations and partitioning behaviour^16^ rather than experimental confirmation. While our study does not resolve this issue, our findings can be evaluated in the context of complementary evidence. For instance, a recent study reported that the partition coefficient of tin between biotite and melt decreases significantly under reducing conditions, which may reflect the higher incompatibility of Sn^II^ compared to Sn^IV^^16^. Yet, biotite, stable only under reducing conditions^18^ where Sn^II^ dominates or is the sole species in the melt^11^, can contain tens to hundreds ppm tin^46,47^. This indirectly suggests that Sn^II^ can be incorporated into trioctahedral mica. Moreover, the presence of mixed oxidation states in muscovite (dioctahedral) and lepidolite (trioctahedral) from the Argemela and Beauvoir RMGs, respectively, supports the capacity of both mica types to host Sn^II^. One can still argue that more Sn^IV^ is incorporated in mica, biasing the estimation of *f*O_2_, which might be partly true, but as emphasised above, previous crystallization^18,38^ and solubility^11^ experiments support our general conclusions. Oxidising condition at the end of the MHT is further supporter by fluid inclusion studies revealing CO_2_ >> CH_4_ in Argemela^48^ and Beauvoir^37^ (see also^49^.

The extent and timing of oxidation might define the scale and the style of tin mineralisation. (i) Early oxidation would lead to tin sequestration within the intrusion as disseminated, sub-economic magmatic mineralisation, as demonstrated for Argemela and Beauvoir. Early magmatic crystallization of cassiterite is a common feature in RMGPs^50,51^ - exemplified at Argemela by the crystallization of Cst_I−II_, also reflected by the marked decrease in tin content from magmatic Ms_I_ to Ms_II_^13,52^ (Fig. S-2a). Biotite and ilmenite, if present and for *f*O_2_ ≤ NNO, are not expected to incorporate significant tin. Although oxidation leads to an increase of tin compatibility, partition coefficients between biotite or ilmenite and melt generally remain below 1^10^. Notably, even if most of the tin is sequestered in cassiterite (and ilmenite), tin hosted in micas remains more susceptible to leaching and redistribution by fluids during subsolidus alteration. Exsolved HCl-bearing magmatic fluids from an oxidised melt would still contain several tens ppm of tin as chloride complexes^49^, which is sufficient for the formation of sub- to economic tin greisen (e.g., Zinnwald; Fig. 5e), given that lowering the HCl activity in the fluid, e.g., via the feldspar-destructive greisenisation reactions or *via* dilution by meteoric water, is a very efficient mechanism for cassiterite deposition^19^. (ii) If less evolved granitic melts follow a similar redox pathway as at Argemela and Beauvoir, the amount of fluid exsolved and fluid focusing will play a critical role for tin concentration, greater than that of magmatic processes. (iii) If no oxidation occurs in the course of granite crystallization or at the MHT - as assumed in the established model^1,2^ but remain to be demonstrated - more tin can comparably be transported in magmatic fluids. However, oxidation due to external parameters/agents, such as for example fluid boiling, mixing or fluid-rock interaction - frequently involved in large-scale tin deposit formation - will limit tin transport and instead result in the crystallization of Sn^IV^-bearing hydrothermal micas and cassiterite in veins and greisens (e.g., Panasqueira, Zinnwald; Figs. 4f and 5e; Table S-4).

Overall, there is more than a single path to a tin deposit. Redox reactions are not always essential for tin transport and concentration, and tin granites and related deposit may result from a variety of redox-driven pathways. Pre-enrichment of the source, fractionation of the granitic melt, the timing at which redox reactions occur in relation with the geodynamical evolution, and the lifespan of the magmatic-hydrothermal activity are all key factors for the formation of high-grade tin deposits.

### Tin as a new redox indicator, limitation and future work

As demonstrated in this study, changes in tin oxidation state can occur in the course of a wide range of processes involved in tin cycle (Figs. 1, 2a and 5), provided that a redox partner is present. In addition, tin redox state can be measured for concentration as low as 30 ppm, tin is present at measurable concentration in a variety of crustal rocks, and redox changes occur in a narrower range of *f*O_2_ compared to Fe^III^/Fe^II^ (e.g., spanning over ~ 5 *f*O_2_ log units in granitic melt^11^ *vs* at least 10–15 for Fe^53^) offering a greater sensitivity. With a lower redox potential, tin can be complementary to Fe to determine *f*O_2_ conditions and redox reactions. It can be particularly important in Fe-poor hydrous granitic melts such as evolved tin-granites as their redox state may not be buffered by Fe^III^/Fe^II^. Instead, equilibria between C-O-H species have been suggested as a potential buffer^43^.

As a limitation and similarly to Fe, the measurement of natural samples of different age raises the question of the preservation of their redox signature. For example, A2-type granitic melts might be more oxidised than those of other “tin-granites” (e.g., dryer source rocks/melt linked to granulitisation; higher Fe^III^/Fe^II^ in ilmenite) contributing to their overall initial Sn^IV^ budget. However, it is difficult to establish with certainty if the general Sn^IV^ signature of Neo-Proterozoic samples from Egypt (Fig. 4e) - the older samples in this study - result from magmatic-hydrothermal processes or if micas were equilibrated by younger events. It is therefore very important to carefully characterise the investigated samples. Though, as stated above, such equilibration can only occur provided that a redox partner is present. Moreover, the different redox states measured in volcanic equivalents to tin granites in this study seem to be related to different processes (Fig. 5c) with no apparent correlation with age (Table S-1).

Overall, very little data are available to constrain precisely redox conditions in tin-granitic systems as opposition to more mafic environments. For example, the Fe^III^/Fe^II^ in minerals (e.g., ilmenite, micas) and systematic experimental calibration of Fe^III^/Fe^II^ in relation with *f*O_2_ in granitic/rhyolitic systems is lacking. Similar work needs to be done for tin and possible fractionation of Sn^II^ and Sn^IV^ due to preferential incorporation in mica with variable composition need to be investigated experimentally. Yet, we believe that this study marks an important step toward the definition of a new redox indicator that might help solving important questions.

## Conclusions

We showed that tin is a useful marker of redox conditions within the continental crust in different geological environments and that tin can be used as a new redox indicator. In the entire process of tin cycle, redox reactions may only be significant, but not essential, during peraluminous magma generation and granite crystallization. Tin ore-deposit formation may result from a variety of redox-driven pathways which may strongly depend on the geodynamic setting. We demonstrated that oxidation occurs earlier than assumed in magmatic-hydrothermal systems, challenging the model established for more than 30 years. This has strong implications on tin ore deposit formation. Tin redox has the potential to become a broadly used tool to help solving major questions involved in Earth’s redox evolution.

## Supplementary Information

Below is the link to the electronic supplementary material.

Supplementary Material 1

Supplementary Material 2

Supplementary Material 3

Supplementary Material 4

Supplementary Material 5

Supplementary Material 6

Supplementary Material 7

Supplementary Material 8

Supplementary Material 9

Supplementary Material 10

## Data Availability

All data generated or analysed during this study are included in the published article and its Supplemental information files (Supplemental Material including Fig. S-1 to S-5, Tables S-1 to S-4).
