# Supplementary material for: Revisiting redox-driven pathways of tin cycle from source to economic deposit
Source: Sci Rep. 2025 Oct 3;15:34476. doi: 10.1038/s41598-025-21389-5 (PMC12494948; doi:10.1038/s41598-025-21389-5)
Supplement: Supplementary file 1 — Supplementary Material 1 [file 41598_2025_21389_MOESM1_ESM.docx]

Revisiting redox-driven pathways of tin cycle from source to economic deposit

**Julie A.-S. Michaud*, Christian Schmidt, Maria A. Naumova**

**Supplemental Text**

**Reference materials and sample preparation**

***Natural and synthetic reference materials***

These include seventeen substances with known oxidation state of tin, including three silicates (malayaite CaSnO[SiO_4_], stokesite CaSn[Si_3_O_9_]·2H_2_O, sørensenite Na_4_SnBe_2_Si_6_O_16_(OH)_4_) and two synthetic silicate glasses (BFGred and BFGox), five oxides (synthetic SnO, synthetic SnO_2_, cassiterite SnO_2_, foordite Sn(Nb,Ta)_2_O_6_, wodginite MnSnTa_2_O_8_), and one hydroxide (mushistonite (Cu,Fe,Zn)Sn(OH)_6_), two synthetic chloride hydrates (SnCl_2_·2H_2_O and SnCl_4_·5H_2_O), three sulphides (herzenbergite SnS, teallite (PbSnS_2_, berndite Sn^IV^S_2_) and metallic tin (Table S-3). In addition, we analysed four samples with unknown Sn^II^-Sn^IV^ ratio (synthetic SnS_2_, stannite Cu_2_FeSnS_4_, franckeite Fe(Pb,Sn^II^)_6_Sn^IV^_2_Sb_2_S_14_), and ixiolite (Ta,Nb,Sn,Mn,Fe)_4_O_8_). Tin-bearing peraluminous glasses (BFG) were synthetized from a natural granite powder from Himalaya^1^ in a one atmosphere furnace under oxidizing conditions (*i.e.* Sn^IV^) and in a one-atmosphere CO_2_-H_2_ (500 and 38.5 ml/min, respectively) mixing furnace under reducing conditions (*i.e.* Sn^II^) at -0.5 log units relative to the Quartz-Fayalite-Magnetite (QFM buffer^2^). Glass powders were placed in a corundum crucible inside a larger platinum crucible to avoid Sn loss by alloying and melted for 2h at 1400°C, finely powdered and melted again for 1h at 1200°C.

***Sample preparation***

***Pellet preparation.*** Grains were hand-picked using a binocular microscope and, if required, identified using Raman spectroscopy, and then crushed in a mortar. Cellulose powder was added and pellets of 5 mm diameter were pressed and enclosed in Kapton tape. If only a few grains were available, these were enclosed in Kapton foil without further treatment.

***Mica.*** Samples were coarsely crushed, mica flakes hand-picked using a binocular microscope and several flakes were enclosed in sticky Kapton tape for each sample. A particular attention was given on avoiding cassiterite crystals if present in a given sample.

**Analytical procedures**

***SEM***

Backscattered electron (BSE) images and element maps were acquired at the Institute of Mineralogy (Leibniz University, Hannover) using a JSM-7610F SEM equipped with a Bruker XFlash 6160 energy dispersive spectrometer (EDS) detector. The detector was set to an accelerating voltage of 15 kV, a working distance between 15 and 20 mm.

***EPMA***

Analyses of major and minor elements on reference glasses were performed at the Institute of Mineralogy (Leibniz University, Hannover) using a JEOL JXA-iHP200F Field Emission Electron Probe Microanalyzer operated under an acceleration voltage and a beam current of 15kV and 10nA, respectively. Glasses synthetized under reducing and oxidizing conditions were measured with an unfocused beam of 12µm to prevent alkalis migration and underestimation (*i.e.* Na and K). All elements were analysed on the peak for 10s and standards include quartz (SiKα), wollastonite (CaKα), kyanite (AlKα), Fe_2_O_3_ (FeKα), Mn_3_O_4_ (MnKα), Orthoclase (KKα) and Jadeite (NaKα). Results and detection limits are shown in Table S-2. Tin was also measured with EPMA (SnO_2_ standard, SnLα) using the same conditions, detection limits being of ca. 0.03 wt% SnO_2_.

***Bulk rock analyses***

The bulk rock analyses were carried out at the SARM (CRPG, Nancy, France; details are summarised in^3^). The Beauvoir rhyolite sample was analysed using an inductively coupled plasma emission spectrometer (ICPOES) iCap6500 ThermoFisher for major elements and an inductively coupled plasma mass spectrometry (ICP-MS) iCapQ ThermoFisher for trace elements. Detection limits, calculated as the average plus 6 times the absolute standard deviation on 100 measurements of blank, are indicated in Table S-2.

***Variation in the peak height of the edge-jump in XANES spectra***

There are four potential reasons for the peak height in the XANES spectra to vary in the samples. (i) A measurement artefact due to self-absorption. Because most of the presented spectra were acquired by X‑ray fluorescence, it was necessary to verify that self‑absorption did not artificially reduce the white line. This was confirmed during data processing by comparison with transmission spectra, allowing this effect to be ruled out. (ii) Influence of data extraction. If all spectra were normalised the same way, noisy spectra - partly due to very low tin concentration but can be avoided using a different detector and/or with longer measurements - show a small edge jump compared to the background and the tilt of the background varies from sample to sample and from spot to spot in a single sample. This can trigger some peak height variations. In our samples, only DRO9 might be affected with the rest of the spectra showing better data quality. (iii) A mixture of Sn^II^ and Sn^IV^. The “addition” of Sn^II^ to Sn^IV^ in a sample would result in a peak height reduction and a shift in the edge position to lower energy such as illustrated in Fig. S-5. This is the case for several of our samples (e.g., Fig. 4a-d). (iv) Different tin neighbours or structure of the sample. As evidenced from the measurement of reference material (Fig. 3; S-4), even for a sample with pure Sn^IV^, a variable in the line shape is possible. Spectra look different for different neighbours or coordination geometries (Fig. S-4) and the shift in the Sn K-edge can be slightly affected (Fig. 3; Table S-3). For this reason, to consider our sample as “mixed state”, we checked that their E0 energy determined at 50% edge jump intensity is below the lowest energy of the reference material for Sn^IV^ (i.e., below 29200.6 eV for SnCl_4_.5H_2_O) and higher than the highest energy of the reference material for Sn^II^ (i.e., above 29198.3 eV for synthetic SnO).

**REFERENCES**

1. Cheng, L. *et al.* Experimental investigation of reactions between two-mica granite and boron-rich fluids: Implications for the formation of tourmaline granite. *Sci. China Earth Sci.* **62**, 1630–1644 (2019).

2. Linnen, R. L., Pichavant, M. & Holtz, F. The combined effects of *f*O2 and melt composition on SnO2 solubility and tin diffusivity in haplogranitic melts. *Geochimica et Cosmochimica Acta* **60**, 4965–4976 (1996).

3. Carignan, J., Hild, P., Mevelle, G., Morel, J. & Yeghicheyan, D. Routine Analyses of Trace Elements in Geological Samples using Flow Injection and Low Pressure On-Line Liquid Chromatography Coupled to ICP-MS: A Study of Geochemical Reference Materials BR, DR-N, UB-N, AN-G and GH. *Geostandards Newsletter* **25**, 187–198 (2001).

**Supplemental figures**

**
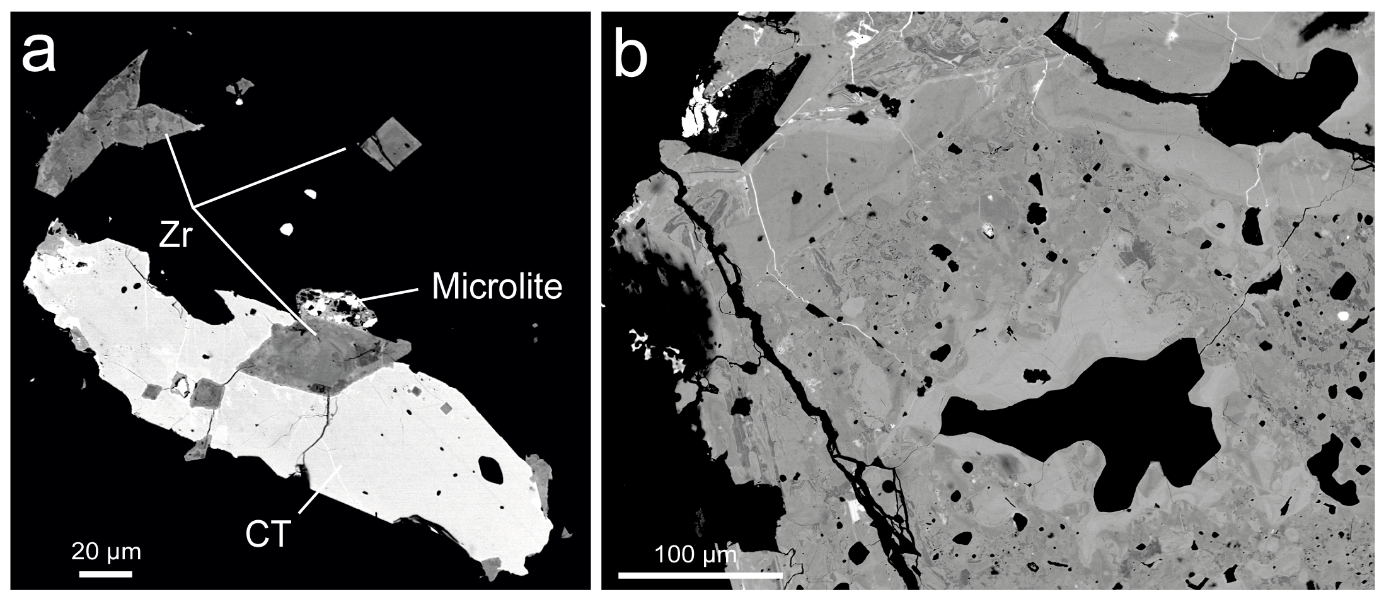
**

**Fig. S-1** Examples of strongly metasomatized zircon (Zr) from the rare-metal granite of Mueilha (a) and a pegmatite from Abu Rusheid (b), Eastern Desert of Egypt. CT: columbite-tantalite.


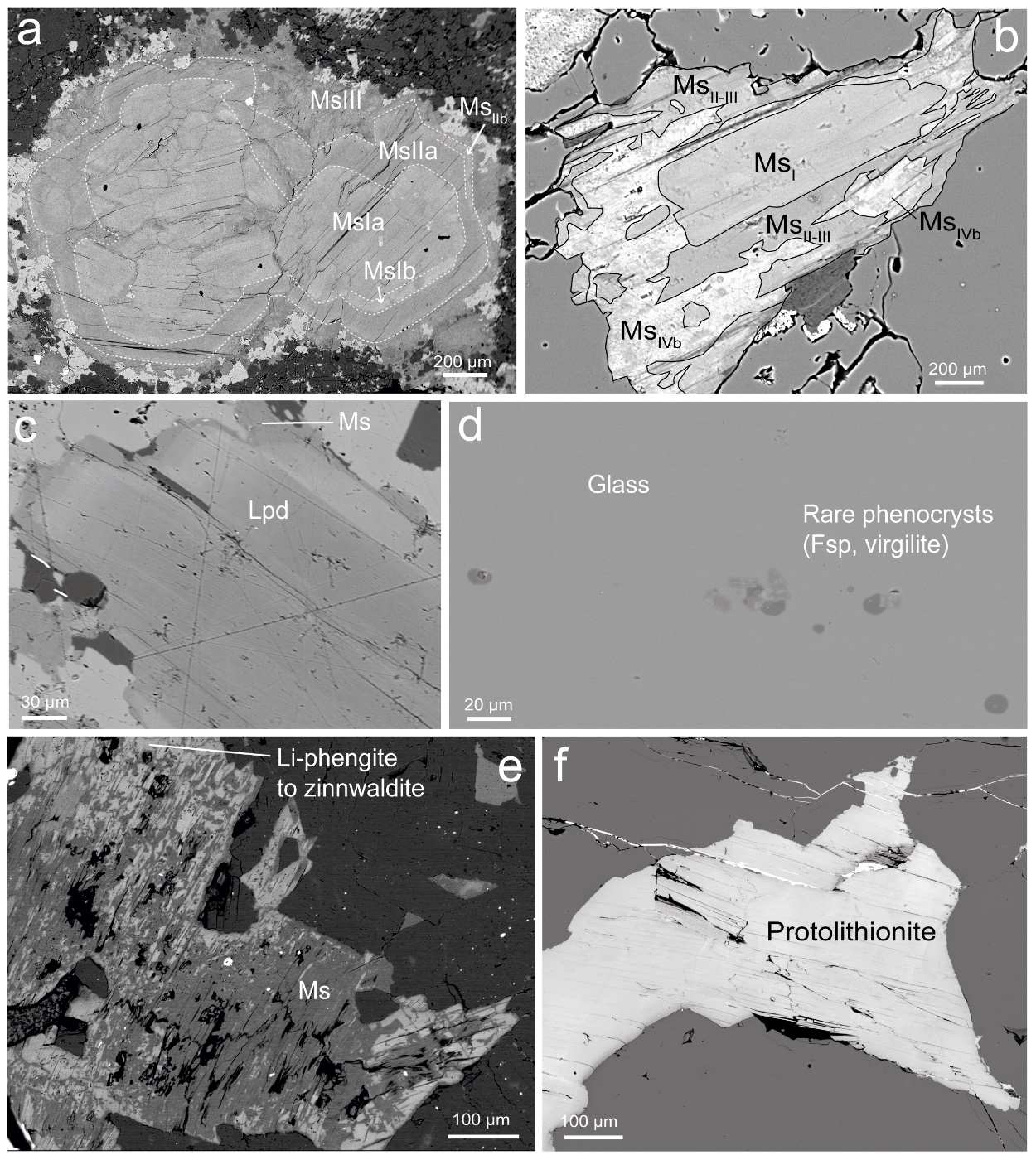
**Fig. S-2** Examples of samples investigated in this study. (a-b) Representative examples of magmatic-early hydrothermal Ms_I-III_ in the granitic facies (a) and subsolidus replacement in the border facies at Argemela (Ms_IV_; pictures from Michaud et Pichavant 2020); (c) example of magmatic lepidolite (Lpd) surrounded by hydrothermal muscovite (Ms) in the B1 facies of the Beauvoir RMG; (d) pristine obsidian glass from Macusani with very few magmatic phenocrysts; (e) extreme subsolidus replacement of magmatic Li-phengite/zinnwaldite by muscovite in the red granite of Mueilha; (f) relatively homogeneous protolithionite from a pegmatitic dyke at Abu Rusheid.


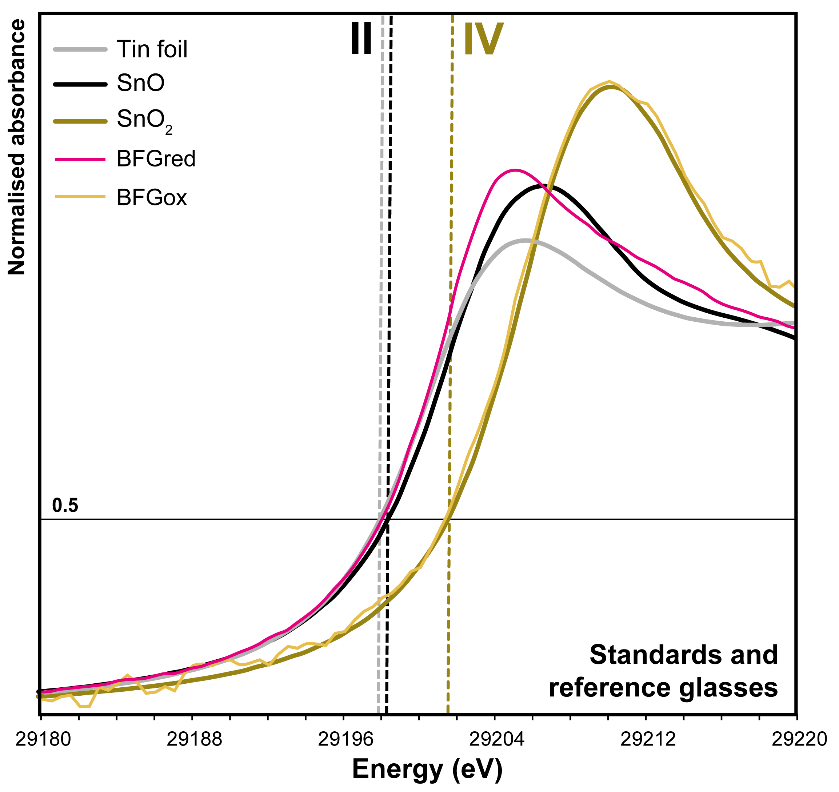


**Fig. S-3** Normalized XANES spectra at the Sn K-edge of the standards Sn^II^O, Sn^IV^O_2_, tin foil Sn^0^, and reference peraluminous glasses synthetized under reducing (BFGred, Sn^II^) and oxidizing conditions (BFGox, Sn^IV^).


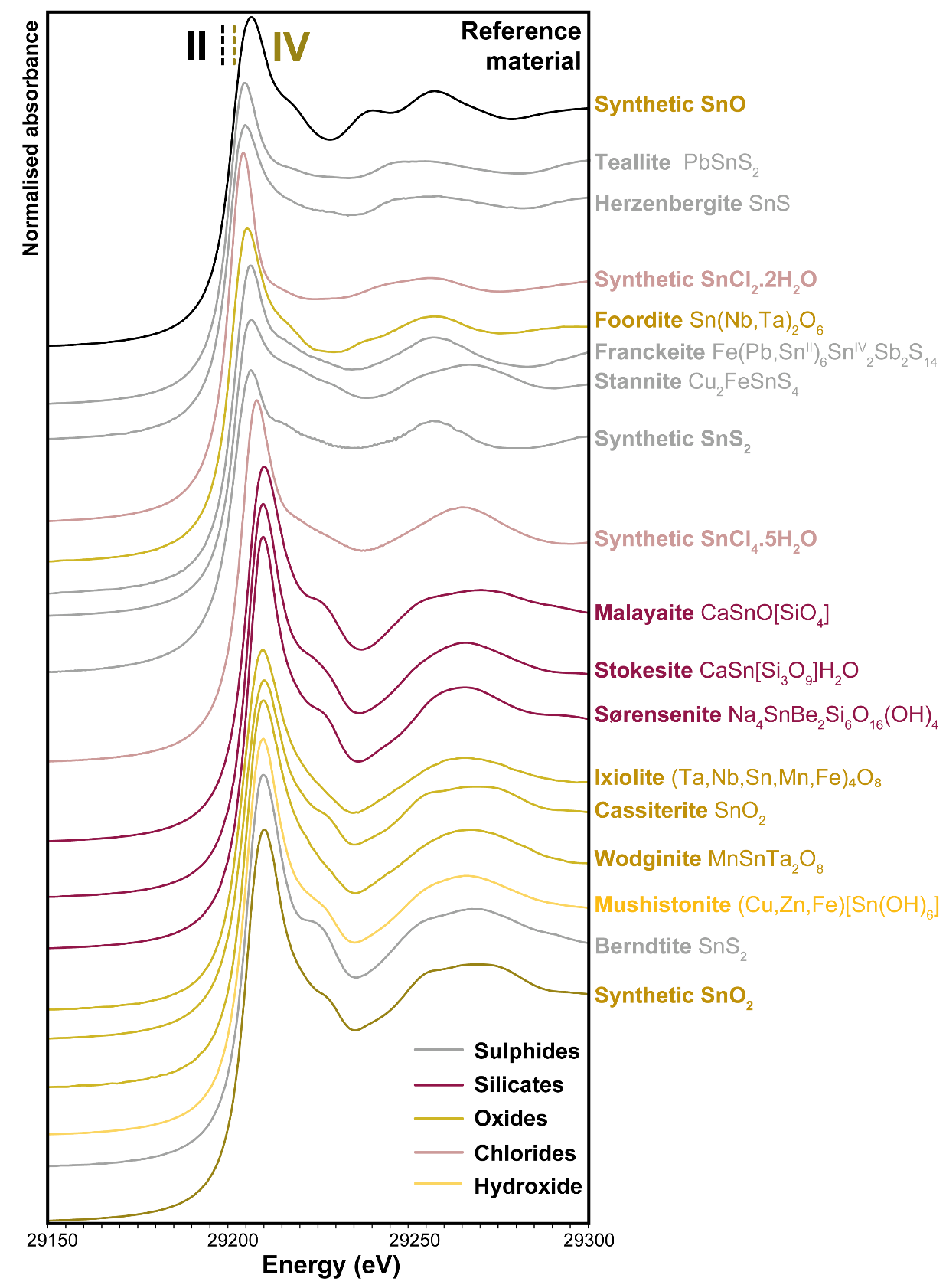


**Fig. S-4** Normalized Sn K-edge XANES spectra of reference materials including sulphides, silicates, oxides, chlorides, and a hydroxide.


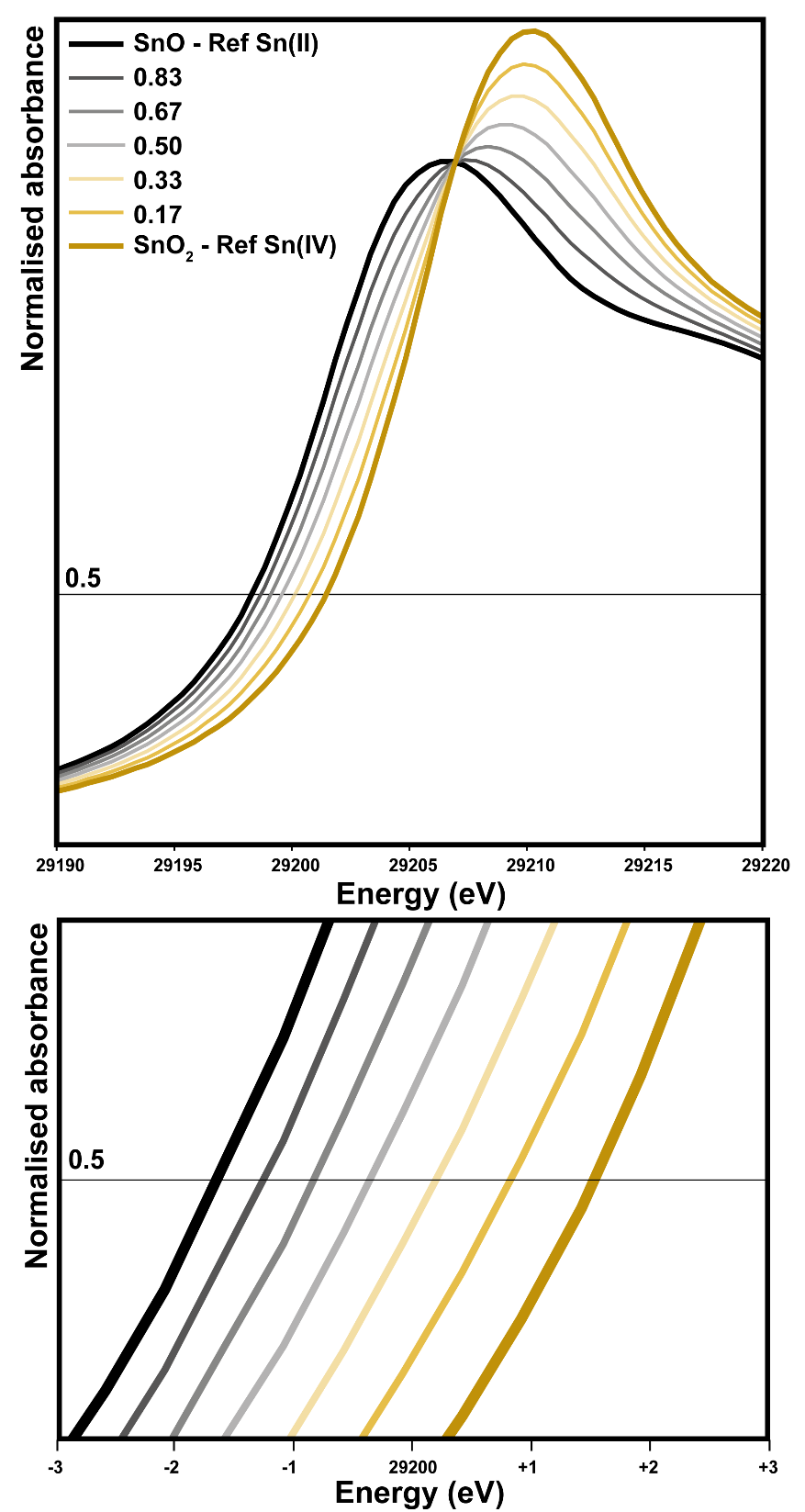


**Fig. S-5** Illustration of the shift in the Sn K-edge accompanied by a change in the peak height of the spectra from the reference SnO_2_ (Sn^IV^) to reference SnO (Sn^II^). Note that this is an ideal case where possible extraction issue and differences in neighbourhood around Sn^II^ and Sn^IV^ or structure of the mineral do not interfere. See discussion in the Supplemental Text for more details.
