# Supplementary figures and images for: Revisiting redox-driven pathways of tin cycle from source to economic deposit
Source: Sci Rep. 2025 Oct 3;15:34476. doi: 10.1038/s41598-025-21389-5 (PMC12494948; doi:10.1038/s41598-025-21389-5)

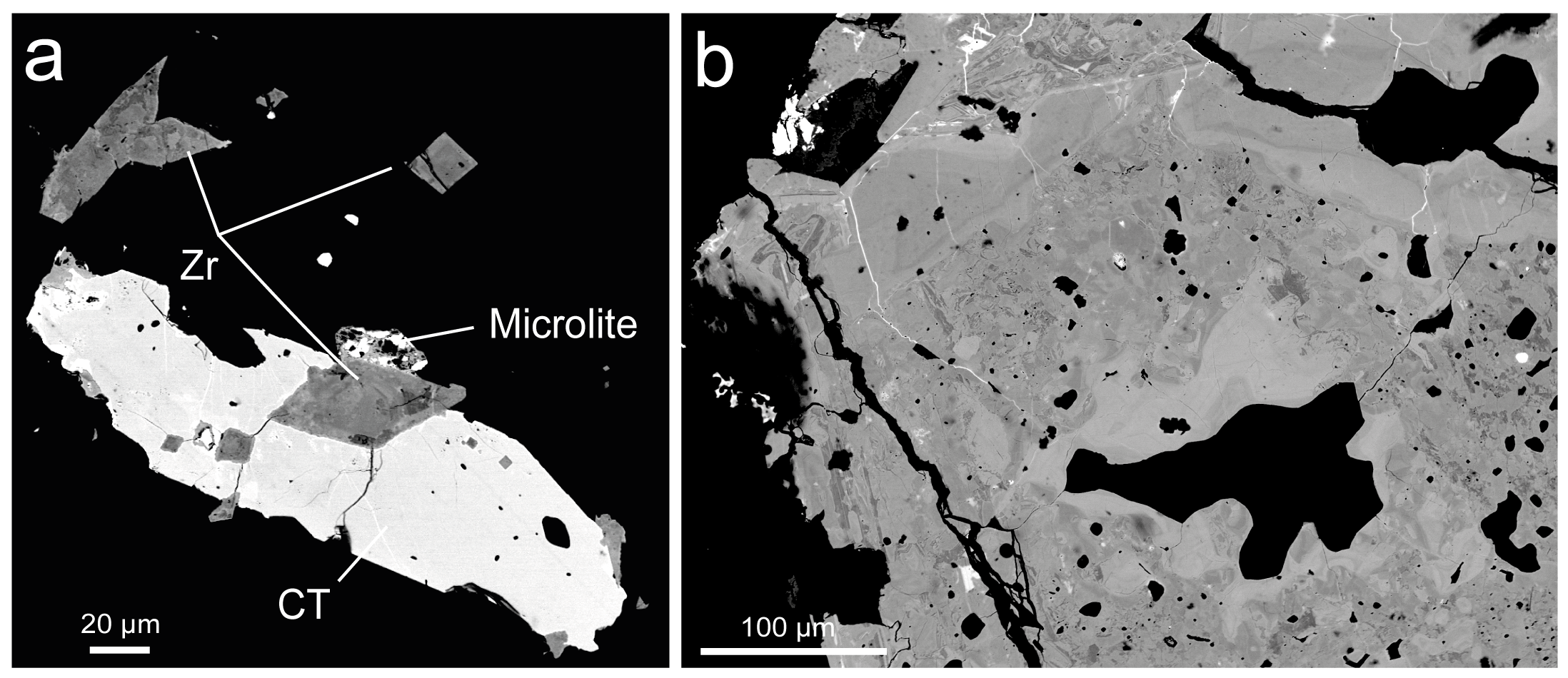

Supplement: Supplementary file 2 — Supplementary Material 2 [file 41598_2025_21389_MOESM2_ESM.tif]

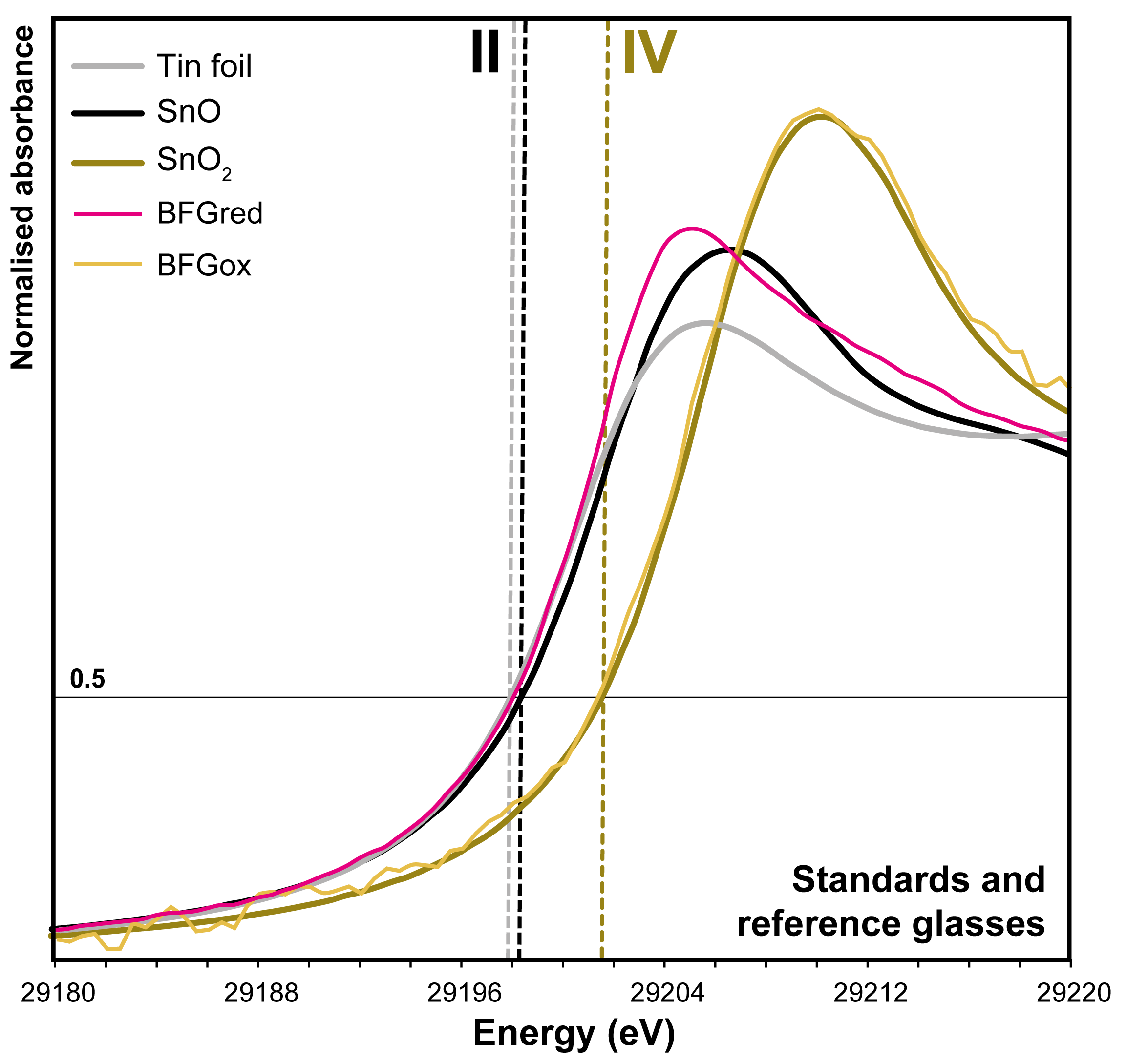

Supplement: Supplementary file 3 — Supplementary Material 3 [file 41598_2025_21389_MOESM3_ESM.tif]

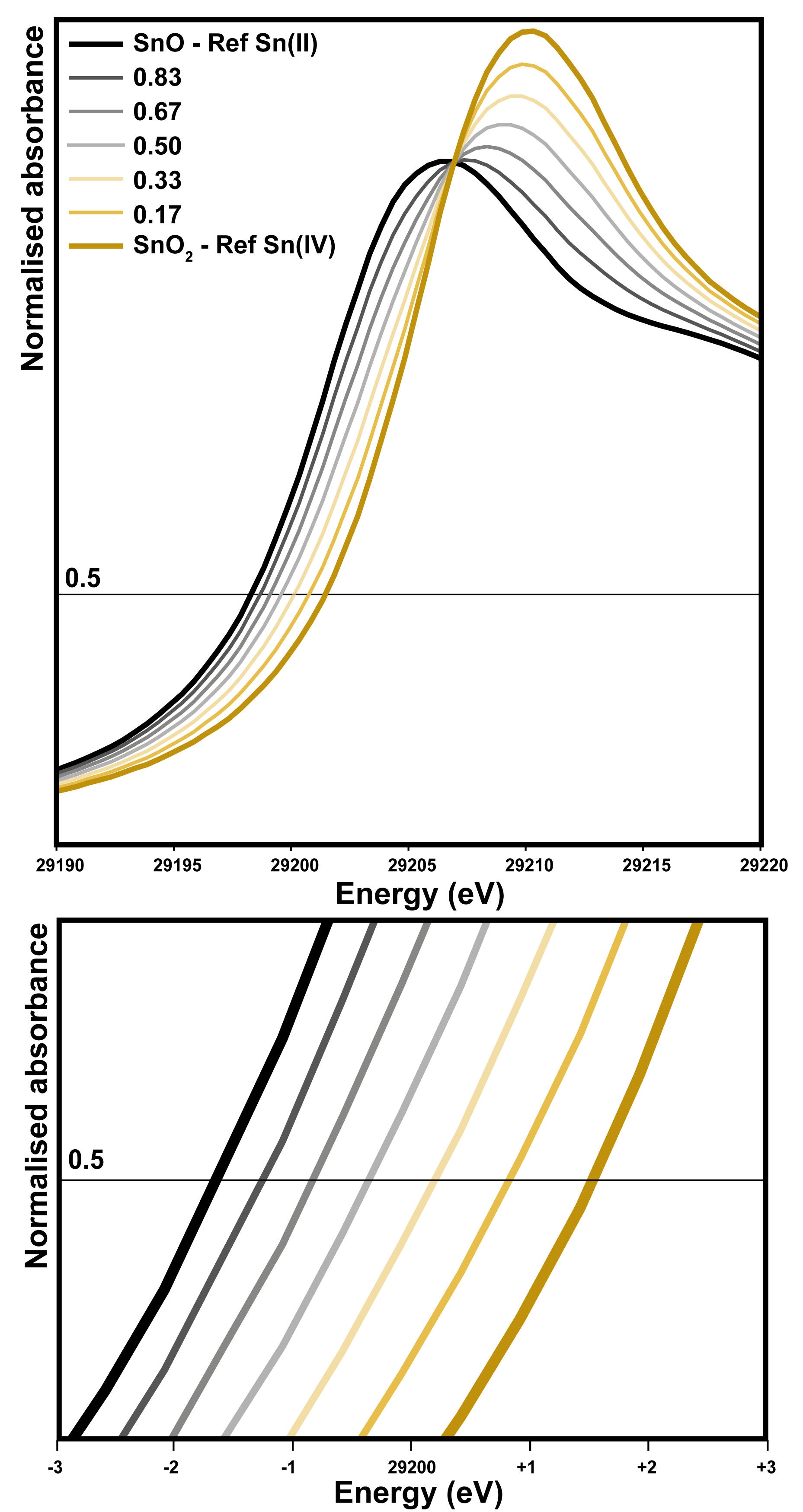

Supplement: Supplementary file 4 — Supplementary Material 4 [file 41598_2025_21389_MOESM4_ESM.tif]

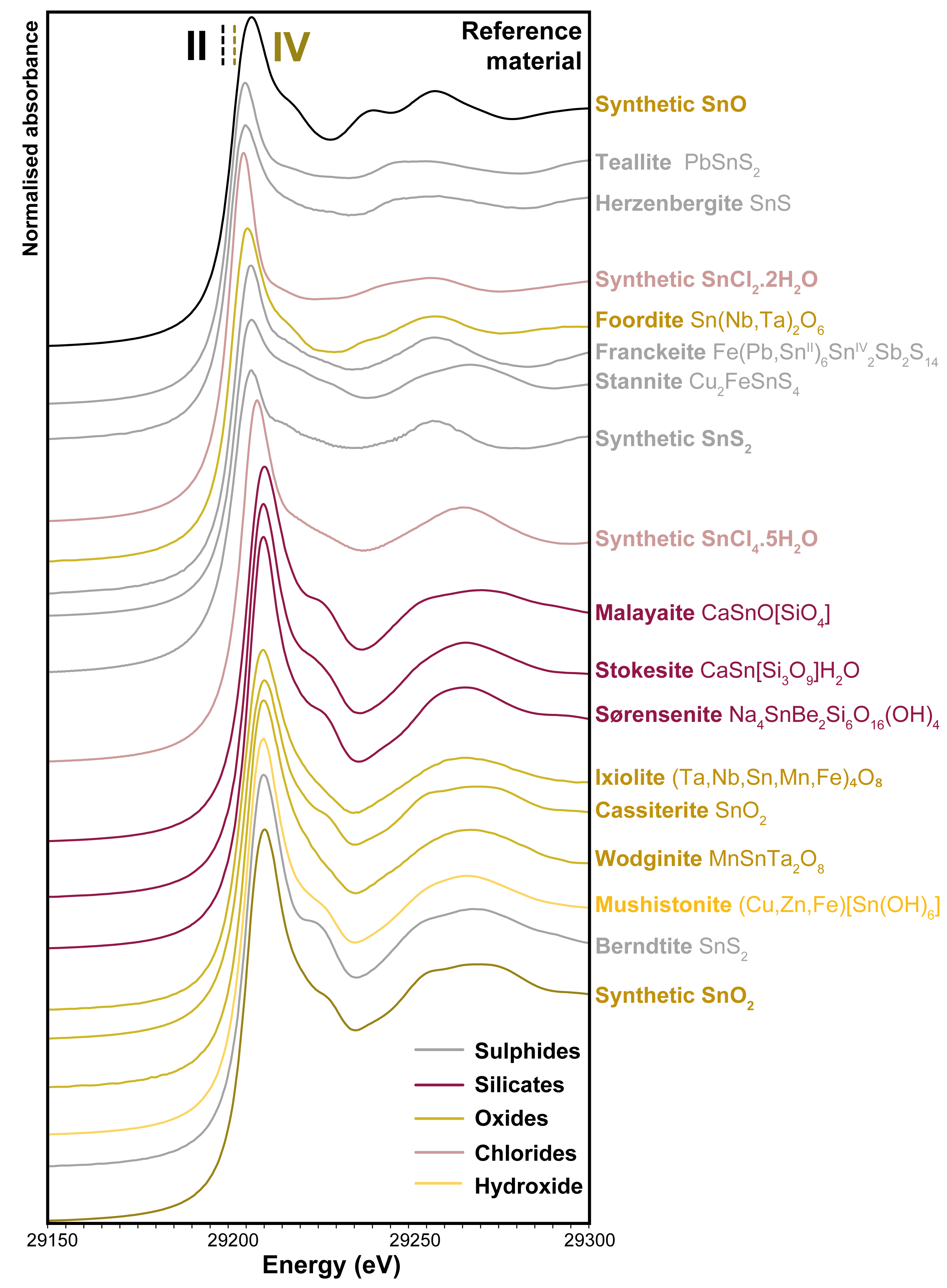

Supplement: Supplementary file 5 — Supplementary Material 5 [file 41598_2025_21389_MOESM5_ESM.tif]

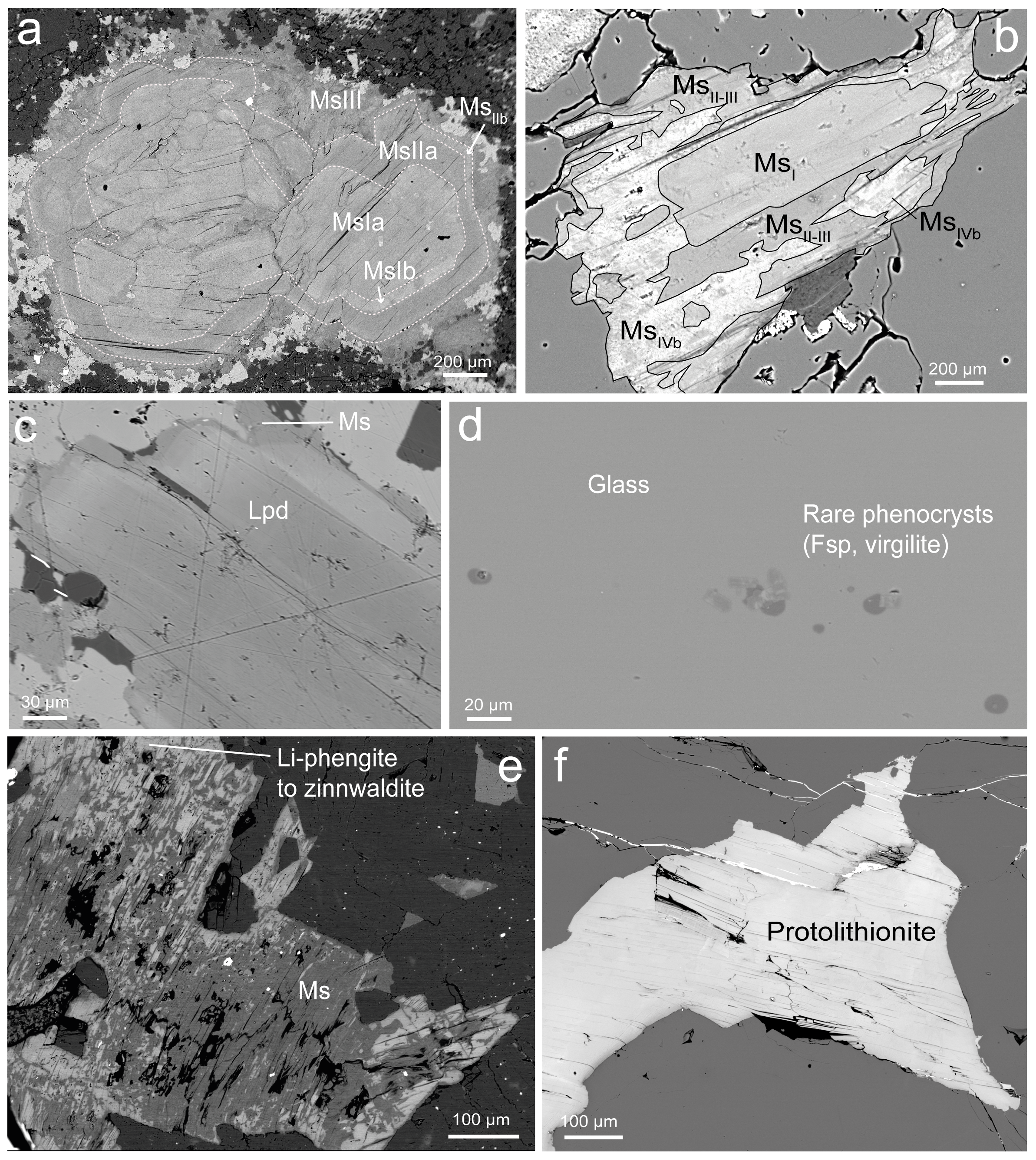

Supplement: Supplementary file 6 — Supplementary Material 6 [file 41598_2025_21389_MOESM6_ESM.tif]
